# Supplementary material for: C-mannosylation supports folding and enhances stability of thrombospondin repeats
Source: eLife. 2019 Dec 23;8:e52978. doi: 10.7554/eLife.52978 (PMC6954052; doi:10.7554/eLife.52978)
Supplement: Supplementary file 1. [file elife-52978-supp1.docx]

**Sequences of used *C. elegans* UNC-5 and *Drosophila*** **Notch constructs**

**pMT‑UNC‑5‑TSR1+2:** MKLCILLAVVAFVGLSLGGWSEWSPWIGTCHVDCPLLRQHAHRIRDPHDVLPHQRRTRTCNNPAPLNDGEYCKGEEEMTRSCKVPCKLDGGWSSWSDWSACSSSCHRYRTRACTVPPPMNGGQPCFGDDLMTQECPAQLCTADSSRGPFEGKPIPNPLLGLDSTRTGHHHHHH 17.42 kDa

**pMT‑UNC‑5‑TSR2:**MKLCILLAVVAFVGLSLGLDGGWSSWSDWSACSSSCHRYRTRACTVPPPMNGGQPCFGDDLMTQECPAQLCTADSTGHHHHHH 7.15 kDa

**pMT‑Notch‑EGF16‑20:** MKLCILLAVVAFVGLSLGKQINECESNPCQFDGHCQDRVGSYYCQCQAGTSGKNCEVNVNECHSNPCNNGATCIDGINSYKCQCVPGFTGQHCEKNVDECISSPCANNGVCIDQVNGYKCECPRGFYDAHCLSDVDECASNPCVNEGRCEDGINEFICHCPPGYTGKRCELDIDECSSNPCQHGGTCYDKLNAFSCQCMPGYTGQKCETNIDDLESRGPFEGKPIPNPLLGLDSTRTGHHHHHH 24.7 kDa

Signal sequence and V5-His Tags underlined.
